# Diagnostic Options and Challenges for Dengue and Chikungunya Viruses

**DOI:** 10.1155/2015/834371

**Published:** 2015-10-05

**Authors:** Stacey K. Mardekian, Amity L. Roberts

**Affiliations:** Sidney Kimmel Medical College at Thomas Jefferson University, 117 South 11th Street, PAV 207, Philadelphia, PA 19107, USA

## Abstract

Dengue virus (DENV) and Chikungunya virus (CHIKV) are arboviruses that share the same *Aedes* mosquito vectors and thus overlap in their endemic areas. These two viruses also cause similar clinical presentations, especially in the initial stages of infection, with neither virus possessing any specific distinguishing clinical features. Because the outcomes and management strategies for these two viruses are vastly different, early and accurate diagnosis is imperative. Diagnosis is also important for surveillance, outbreak control, and research related to vaccine and drug development. Available diagnostic tests are aimed at detection of the virus, its antigenic components, or the host immune antibody response. In this review, we describe the recent progress and continued challenges related to the diagnosis of DENV and CHIKV infections.

## 1. Introduction

Dengue virus (DENV) and Chikungunya virus (CHIKV) are single-stranded, positive-sense RNA viruses. DENV belongs to the family Flaviviridae and genus* Flavivirus* of which there are 5 known serotypes (DENV1–5). CHIKV belongs to the family Togaviridae and genus* Alphavirus* of which there are 3 known strains (Asian-West African; East-Central; South African) [1]. The genome of each virus is approximately 11 kb in length [1, 2]. The DENV genome encodes three structural (C, prM, and E) and seven nonstructural (NS1, NS2B, NS3, NS4A, NS4B, and NS5) proteins [3]. The CHIKV genome encodes three structural (C, E1, and E2) and four nonstructural (nsP1–4) proteins [1].

Both viruses are arthropod-borne viruses (arboviruses) sharing a common vector: mosquitos of the* Aedes* genus, specifically* A. aegypti* and* A. albopictus* [4]. Both viruses circulate in similar geographic regions. In nonendemic regions, travel-associated infections are an important consideration for patients with a recent travel history who present with fever. Concurrent infection with both viruses, transmitted from either two different mosquitos or one dually infected mosquito, is possible [5, 6]. For DENV, transmission has also been reported to occur via infected blood products, organ donation, and prenatal and/or perinatal vertical transmission [7].

While DENV and CHIKV present similarly as an acute febrile illness, these two viruses have vastly different management strategies and outcomes. The majority of CHIKV infections are self-limiting with chronic joint disease being the most common long-term outcome, and fatality is exceedingly rare. Nonsteroidal anti-inflammatory drugs (NSAIDs) are the mainstay treatment for CHIKV, but NSAIDs should be avoided until DENV is confidently ruled out, as NSAIDs are contraindicated in DENV infection [8]. DENV is likewise commonly a self-limiting illness, yet this diagnosis necessitates stricter monitoring due to the potential for more significant morbidity and mortality. Infection with one serotype of DENV confers lifelong immunity to that particular serotype but only short-term immunity to the other serotypes; subsequent infections with a different serotype increase the risk of severe complications [7].

## 2. Epidemiology

The majority of DENV and CHIKV infections affect people residing in endemic areas, which include most of the tropical and subtropical regions in the world. Many of these areas serve as popular tourist destinations and, consequently, dengue-related infections have recently surpassed malaria and gastrointestinal infections as the most common cause of fever among travelers [23]. The major endemic regions include Southeast Asia, the Western Pacific, the Eastern Mediterranean, Africa, and the Americas [9]. Specific countries with cocirculation and coinfections of DENV and CHIKV include India, Sri Lanka, Gabon, Cameroon, Madagascar, Indonesia, Singapore, and Thailand [24]. In the United States, autochthonous outbreaks of DENV have been reported in Hawaii and along the Texas-Mexico border, and outbreaks of both DENV and CHIKV have recently occurred in southwest Florida [6, 25].

## 3. Clinical Presentation

These two viruses share a similar geographic distribution; unfortunately, their clinical manifestations also show substantial overlap. The typical incubation periods for DENV and CHIKV are 4–7 days and 3–7 days, respectively [4]. Patients infected with either virus typically present with acute onset of fever, myalgia, and headache, and some patients experience a maculopapular rash and/or gastrointestinal symptoms [4, 6].

A classification scheme for DENV, put forth by the World Health Organization (WHO) in 2009, includes criteria for probable dengue and severe dengue [9]. Most DENV infections are either asymptomatic or mild and self-limited, but there are “warning signs” that may suggest which patients may progress to severe disease and require stricter medical management [9]. Severe dengue may manifest as significant plasma leakage, hemorrhagic complications, and/or severe organ impairment, so early recognition of DENV infection is imperative [9]. Compromising the sensitivity of the WHO classification scheme is the fact that patient age influences the type and severity of symptoms; Low et al. found that fewer older adults reported symptoms of myalgia and arthralgia, as well as mucosal bleeding, which is one of the primary “warning signs” [26].

The clinical course for CHIKV is likewise typically mild and self-limited. The hallmark presentation of CHIKV is a bilateral migratory arthralgia, often intense, affecting mainly the small joints of the extremities [1, 4]. However, most children with CHIKV report only mild arthralgia [8]. The major long-term complication is persistence of joint pain and stiffness, which may last years after resolution of the initial infection [1]. Rarely, CHIKV infection is associated with neurologic, ophthalmologic, and hemorrhagic disease [4, 5].

While neither infection possesses a defining clinical feature, there are suggested trends in the symptomatology and complete blood count (CBC) results that may help differentiate between the two infectious processes. It is suggested that, at initial presentation, significantly more DENV patients have thrombocytopenia (platelets < 100 × 10^9^/L) and associated minor bleeding complications such as petechiae and nose bleeds, while patients with CHIKV are more likely to have arthralgia. Leukopenia is common to both infections at initial presentation but tends to be more pronounced in DENV patients; CHIKV patients tend to have higher white blood cell (WBC) counts (>3.6 or 5.0 × 10^9^/L according to two separate authors) than DENV patients [4, 6, 8]. During the course of illness, DENV patients are more likely to have abdominal pain and the CBC will demonstrate leukopenia, neutropenia, and thrombocytopenia that is more frequent and more pronounced than in CHIKV patients. In contrast, CHIKV patients may show a shorter duration of fever, conjunctivitis, acute arthritis, and more prominent arthralgia affecting multiple joints [6]. While these trends in clinical findings may be helpful, they are neither specific nor consistent enough to be considered diagnostic.

Unfortunately, there is no single clinical or laboratory marker available for distinguishing DENV or CHIKV infection from each other or from other acute febrile illnesses. Therefore, both of these viruses must be initially included in the differential diagnosis for a patient with suspicious clinical symptoms who is living in or returning from travel to an endemic area. Clinical features can serve, at best, as a guide for favoring one virus over the other, as patients may present atypically, either by lacking the “classic” signs or symptoms as mentioned above, or by presenting in an uncharacteristic manner. Laboratory diagnostic tests are thus essential for accurate identification of the causative virus.

## 4. Methods for Diagnosis

A wide variety of laboratory diagnostic methods are available to aid in the diagnosis of DENV and CHIKV infections. The premise of these tests is detection of the virus, viral components (antigens or nucleic acid), or the host immunologic response to the virus [10]. Therefore, selection and interpretation of testing depends on the kinetics of viremia and antibody response, which differ between primary and secondary infections. Other factors influencing test choice include the purpose of testing and availability of resources. Each type of test offers unique advantages and disadvantages, and a combination of tests may be employed in order to increase diagnostic confidence. For a summary of available tests for DENV and CHIKV infection, see Tables 1 and 2, respectively.

### 4.1. Overview of Currently Available Tests

The acute febrile phase of infection corresponds to the period of viremia, which lasts typically from 5 days after onset of fever for both DENV and CHIKV. During this time, diagnosis rests on isolation of the virus, viral RNA, or viral antigen from the specimen. Isolation of DENV or CHIKV can be performed via mosquito inoculation or cell culture; CHIKV isolation can also be accomplished by intracerebral inoculation of mice [16]. Virus may be recovered from serum, plasma, whole blood, or tissues collected at autopsy. Mosquito inoculation is the most sensitive isolation method but is impractical for routine diagnosis due to the highly specialized requirements and high maintenance costs [3]. Cell culture is in wider use, with preference given to the mosquito cell line C6/36 (cloned from* A. albopictus*) or AP61 (cloned from* A. pseudoscutellaris*) [9, 16]. Other less sensitive options include mammalian cell cultures such as Vero, LLC-MK2, and BHK-21 [3]. The resultant virus isolate may be further characterized during subsequent* in vitro* studies, such as genome sequencing, virus neutralization, and infection studies [3]. Virus isolation is highly specific and has a theoretical detection limit of a single viable virus, although, in practice, the sensitivity is only approximately 40.5% in cell line-based virus isolation. It also requires highly trained operators, a dependence on sample integrity and a short viremia period, thus providing a narrow window of opportunity from illness onset. Virus isolation followed by an immunofluorescence assay for confirmation requires days to weeks [9, 16]. Therefore, despite its advantages, this approach is not widely used in routine diagnostic laboratories and may serve more use in surveillance purposes. A more recent development in viral isolation is described by Patramool et al., who used anionic polymer-coated beads to isolate DENV and CHIKV [27]. This may prove a useful strategy to monitor the status of circulating mosquitos in regions at risk for outbreaks with these arboviruses. Compared to traditional isolation techniques, this method provides reduced cost, good sensitivity, and rapidity, which is conducive to simultaneous analysis of a large number of samples [27].

Compared to virus isolation, viral nucleic acid detection techniques performed on acute-phase specimens offer better sensitivity with a much more rapid turnaround time. Viral nucleic acid can be detected for a few additional days beyond the period of viremia. Detection of viral nucleic acid can be accomplished by reverse transcriptase polymerase chain reaction (RT-PCR), real time RT-PCR, or isothermal amplification methods. All of these methods involve three basic steps: viral RNA extraction, amplification, and detection and characterization of the amplified product [9]. There is a wide variety of specimen types that can be tested with RT-PCR, including blood, serum, plasma, and fresh or formalin-fixed paraffin-embedded tissues. For DENV, urine and saliva have been found to be suitable specimen types as well [3]. Testing urine samples by real-time RT-PCR provides a larger window of detection that extends well past the viremia period; DENV RNA may be detected in urine up to day 16, compared to day 8 for blood specimens [28]. The ability to test urine and saliva is advantageous in patients for whom blood samples are difficult to obtain, such as in newborns and patients with hemorrhagic syndromes [14].

RT-PCR using primers designed for structural and nonstructural domains has been found to be useful in the rapid diagnosis of CHIKV. The combination of RT-PCR/nested PCR has proved efficient for specific detection and genotyping of CHIKV. Loop-mediated isothermal amplification (LAMP) assays can be rapidly carried out at a single temperature in a water bath, with visually detectable results, and comparable sensitivities to conventional PCR [17].

Detection of viral antigens is another diagnostic methodology available for DENV infection. Nonstructural protein 1 (NS1) antigen is a highly conserved glycoprotein produced during the virus replication process, and a soluble form of NS1 accumulates in high concentrations in the serum of patients with both primary and secondary DENV infections [29, 30]. Several commercial assays, consisting of both rapid tests and enzyme-linked immunosorbent assay (ELISA) kits, are available for the detection of the NS1 antigen. Serum is the most common sample type. DENV NS1 can also be detected in urine samples during the acute phase of DENV infection, which provides an opportunity for the development of a rapid noninvasive test [11]. Lastly, NS1 antigen may be detected in the cerebrospinal fluid (CSF) of patients with neurological symptoms [12]. A downfall is that these tests do not differentiate between dengue serotypes, as NS1 is highly conserved by all serotypes. Additionally, these tests are most successful during the acute phase of illness and lose sensitivity once the period of viremia ends. The sensitivity of NS1 has also been found to be lower in DENV secondary infections, which is thought to be due to assay interference by anti-NS1 antibodies which are present more frequently in secondary infections [10, 29]. An antigen-based commercial detection assay is not widely available for CHIKV, and the ones described thus far in the literature have unclearly established performance characteristics [21, 22].

After the period of viremia, the methods described thus far become much less sensitive for diagnosis. At this point, the best diagnostic strategy entails detection of antibodies indicative of host immune response to the virus. However, the caveat is that individuals in endemic areas often have immunologic levels to these viruses. Serologic methods include ELISA, indirect immunofluorescence assays (IFA), hemagglutination inhibition (HI), and microneutralization (MNt) [1]. ELISA and IFA are rapid and sensitive techniques for detecting virus-specific antibodies and can distinguish between IgG and IgM. For techniques that cannot make this distinction (HI and MNt), it is required to compare paired serum samples (acute and convalescent phases) to establish recent infection.

For DENV, serologic methods are most commonly employed, in particular IgM capture ELISA [4]. IgM antibodies are detectable in 50% by days 3–5 after onset, 80% by day 5, and 99% by day 10 after initial symptoms. They may persist for months; hence DENV IgM antibodies are a reliable marker of recent but not necessarily acute infection [29]. IgG antibody response develops a few days after the onset of IgM antibodies, and IgG may persist for many years [29]. Serologic confirmation of infection requires demonstration of a fourfold rise in antibody titer between acute and convalescent phase sera, or by demonstration of IgM antibodies specific for the virus [16]. Patterns of antibody response differ between primary and secondary infections, with primary dengue invoking stronger and more specific IgM response than in secondary, which have stronger and more rapid IgG response. Prior vaccination against other* Flavivirus* (Japanese encephalitis virus; Yellow-fever virus) or prior infection with nondengue flaviviruses (including West Nile) can potentially influence antibody responses measured in some assays [4]. The recent introduction of rapid diagnostic kits that offer combined detection of NS1 and IgM/IgG antibodies was an effort to create a point-of-care test with better performance characteristics [13]. Evaluation of some of these combined tests has revealed diagnostic sensitivity of 89–93% and specificity of 75–100% [3, 13].

A combination of molecular and IgM antibody detection assays is recommended for diagnosis of CHIKV infection. Some advocate adopting an algorithmic approach, wherein the IgM capture ELISA is used as an initial screening tool followed by the use of rapid molecular assays in CHIKV IgM negative samples, to facilitate rapid diagnosis during outbreaks [18].

### 4.2. Simultaneous Testing for DENV and CHIKV

Because infection with DENV and CHIKV should be on the differential diagnosis together at the initial patient presentation, tests that screen for these viruses simultaneously are preferred to test for them separately. CHIKV and DENV are not readily differentiated serologically due to cross-reactivity of their serocomplexes, so there is a reliance on molecular detection methods for this purpose [19]. A one-step duplex conventional RT-PCR assay for distinguishing DENV and CHIKV has been reported [20]. Saha et al. developed a highly sensitive and specific, rapid one-tube duplex RT-PCR assay which provides a result within 110 minutes [19]. Two authors have described a one-step multiplex real-time RT-PCR assay that can simultaneously detect and quantitate RNA for all DENV serotypes and CHIKV. Cecilia et al. report a sensitivity of 100% for DENV and 95.8% for CHIKV, while the specificity was 100% for both viruses when compared to conventional RT-PCR [24]. Pongsiri et al. report an assay sensitivity of 97.65% and specificity of 92.59% when compared to conventional RT-PCR [31]. Real-time reverse transcription-loop-mediated isothermal amplification (RT-LAMP) is a sensitive alternative to real-time PCR for use in field applications [18]. A RT-LAMP method has been described in which a reverse transcription and amplification was designed in one step with two tubes under the same reaction conditions for the rapid identification and quantitative detection of RNA for CHIKV and DENV, respectively [32]. This assay has a sensitivity of 100% and specificity of 95.25%. The LAMP reaction can be ended within one hour under isothermal conditions and does not require sophisticated instruments, making this method adaptive to field diagnosis. Additionally, the use of a turbidimeter allows for quantitative detection of viral load [32]. For RT-PCR assays described above, the one-step process reduces the chance of contamination and there is lack of cross-reactivity between related* Flavivirus* groups and DENV [19].

### 4.3. Sending Out Samples

Within the United States, CHIKV testing is performed at the Centers for Disease Control and Prevention (CDC), a limited number of select state health departments, and one commercial laboratory. The CDC's Arbovirus Diagnostic Laboratory at the Division of Vector-Borne Diseases (DVBD) is located in Fort Collins, CO. Test results are normally available 4 to 14 days after specimen receipt, but reporting times may be longer during summer months when arbovirus activity increases. Initial serological testing is performed using IgM capture ELISA and IgG ELISA. If the initial results are positive, further confirmatory testing is performed which may delay the reporting of final results. All results are sent to the appropriate state health department.

The CDC Dengue Branch, located in San Juan, Puerto Rico, provides DENV testing free of charge to submitting physicians and state and private laboratories. A “Dengue Case Investigation Form” must accompany the specimen. One potentially problematic issue with sending samples to this laboratory is that an international shipping license is required. Another challenge, especially for underdeveloped countries, is specimen preservation during shipment. The CDC recommendation is that the serum specimen is frozen immediately after separation and sent on dry ice, or alternatively kept refrigerated and sent in cold packs.

### 4.4. Future Test Developments

Other diagnostic methodologies may be available for future use in the laboratory diagnosis of DENV and CHIKV infection. One technique becoming an increasingly popular serological option in arbovirology is microsphere-based immunoassay (MIA). This technology is based on detection by flow cytometry of antigen or antibody attached to microspheres or beads. This is a much more rapid test than MAC-ELISA and also has the potential for performance in multiplex [33]. Similarly, microarray technology, which focuses on detection of nucleic acid fragments corresponding to different pathogens, is useful to screen a sample for the many pathogens on a wide differential diagnosis for infectious symptoms in a given region [10]. Finally, mass spectrometry could be applied to this field of diagnosis, proving especially useful in determining viral serotypes and genotypes during an outbreak [9].

## 5. Conclusion

Confirmation of DENV or CHIKV infection requires laboratory diagnosis. Molecular assays are more sensitive for diagnosis in the early stages of illness (2–5 days after onset) when antibodies are not detected. However, in the later stages of illness, the sensitivity of molecular methods decreases due to the onset of a brisk immune response and corresponding reduction in viral load. At this stage, the IgM ELISA is a more sensitive diagnostic test.

An ideal diagnostic test meets certain key criteria: affordability by those at risk of infection, specificity, sensitivity, ease of use, rapid results, little reliance on equipment, and delivery to those in need [29]. The ideal test should also be part of a multiplexed assay for other pathogens causing acute undifferentiated fever, such as malaria [17]. Progress for DENV and CHIKV diagnostic testing has been made. Generally, tests with high sensitivity and high specificity require more complex technologies and technical expertise, while rapid tests may sacrifice sensitivity and specificity for the advantages of speed and ease of performance. It is difficult to find a balance between accessibility of a diagnostic method and the confidence in the test results. Antigen detection assays seem most promising for rapid and early diagnosis in rural areas. In this regard, development of DENV diagnostic tests is ahead of those for CHIKV, but clearly both of these arboviruses are important causes of disease in their shared endemic regions and in travelers to these areas.

## Figures and Tables

**Table 1:** Diagnostic tests for DENV infection.

Premise of test	Method	Sample types	Sensitivity (%)	Specificity (%)	Advantages	Disadvantages	References
Detection of virus	Virus isolation	Serum, plasma, whole blood, and fresh or FFPE tissues	71.5–84.2 (mosquito inoculation); 40.5 (cell line-based)	100	Greatest specificity Allows for further characterization of isolate	Technical, laborious Variable sensitivity Narrow window of detection (viremic period)	[3, 9, 10]

Detection of viral antigen	NS1 detection via ELISA	Serum, urine, and CSF	54.2–93.4 (serum); 73.9–76.9 (urine) 50 (CSF, if neurological symptoms)	71–80 (serum); 100 (CSF, if neurological symptoms)	Early diagnosis Rapid tests available	Does not differentiate between serotypes Lower sensitivity in secondary infections	[11–13]

Detection of viral nucleic acid	RT-PCR	Serum, plasma, whole blood, fresh or FFPE tissues, urine, and saliva	48.4–98.2	100	Rapid turnaround time Multiplex available (can identify all serotypes from single sample; less potential for contamination)	Expensive reagents and specialized equipment	[3, 9, 14]
	Real-time RT-PCR		58.9–100	100			
	Isothermal amplification methods (NASBA, LAMP)		98.5	100	Does not require specialized equipment (i.e., thermocyclers)		

Detection of host antibody response	MAC-ELISA	Serum	61.5–99	79.9–97.8	Detection of IgM is considered diagnostic	Cross-reactivity among serotypes (not serotype-specific) and with other flaviviruses (false-positives)	[3, 9, 14, 15]
	IgG ELISA				Can distinguish primary from secondary infection using paired sera	Later diagnosis (need postconvalescent sample)	
	IgM/IgG ratio				Distinguishes between primary from secondary infection	Later diagnosis	
	IgA	Serum and saliva	93 (serum); 70–92 (saliva)	88 (serum); 97 (saliva)	Option for testing saliva (easier sample to obtain) Better sensitivity and specificity in secondary infection	Lower sensitivity in primary infection	

**Table 2:** Diagnostic tests for CHIKV infection.

Premise	Diagnostic method	Sample types	Sensitivity (%)	Specificity (%)	Advantages	Disadvantages	References
Detection of virus	Virus isolation (*in vivo* or *in vitro*)	Serum, plasma, whole blood, and fresh or FFPE tissues	Variable	100	Highly specific	Technical, laborious Requires biosafety level 3 containment May take 1-2 weeks	[1]

Detection of viral antigen	ELISA or immunochromatographic assay (ICA)	Serum and CSF	85 (serum) 80 (CSF)	89 (serum) 87 (CSF)	Early diagnosis	Commercial assays not widely available Requires biosafety level 3 containment	[16, 17]

Detection of viral nucleic acid	RT-PCR	Serum and dried blood spots	100	Up to 100	Highly sensitive and specific Rapid turnaround time Multiplex available	Expensive reagents and specialized equipment	[13, 16, 18–20]
	Real-time RT-PCR		100	Up to 100	Multiplex available	Expensive reagents and specialized equipment	
	Isothermal amplification methods (RT-LAMP)		100	95.25	Does not require specialized equipment (i.e., thermocyclers)		

Detection of host antibody response	ELISA	Serum CSF	IgM: 17 (serum); 48 (CSF) IgG: 45 (serum); 63 (CSF)	IgM: 95 (serum) IgG: 53 (serum)	Widely available Relatively cheaper and easier to perform Rapid bedside tests are available	Possible cross-reactivity with other alphaviruses Elevated IgM does not distinguish recent past infection from acute infection	[4, 16, 17, 20–22]
	IFA	Serum	85–97	90–98	Sensitive and specific Commercially available	Lack the ability to quantify antibodies, are subjective, and require special equipment and training	
	PRNT	Serum			Very specific for alphaviruses; gold standard for confirmation of serologic test results	Requires the use of live virus (requires Biosafety level 3 containment)	
